# Assessing Strategies Against Gambiense Sleeping Sickness Through Mathematical Modeling

**DOI:** 10.1093/cid/ciy018

**Published:** 2018-06-01

**Authors:** Kat S Rock, Martial L Ndeffo-Mbah, Soledad Castaño, Cody Palmer, Abhishek Pandey, Katherine E Atkins, Joseph M Ndung’u, T Déirdre Hollingsworth, Alison Galvani, Caitlin Bever, Nakul Chitnis, Matt J Keeling

**Affiliations:** 1 Zeeman Institute for Systems Biology and Infectious Disease Epidemiology Research, Coventry, United Kingdom; 2 School of Life Sciences, University of Warwick, Coventry, United Kingdom; 3 Yale School of Public Health, Yale University, New Haven, Connecticut; 4 Department of Epidemiology and Public Health, Swiss Tropical and Public Health Institute, Switzerland; 5 University of Basel, Switzerland; 6 Institute of Disease Modeling, Bellevue, Washington; 7 Department of Infectious Disease Epidemiology, Faculty of Epidemiology and Population Health, United Kingdom; 8 Centre for Mathematical Modelling of Infectious Diseases, London School of Hygiene and Tropical Medicine, United Kingdom; 9 Foundation for Innovative New Diagnostics, Geneva, Switzerland; 10 Mathematics Institute, University of Warwick, Coventry, United Kingdom

**Keywords:** gambiense human African trypanosomiasis, HAT, mathematical modeling, intervention effectiveness, elimination

## Abstract

**Background:**

Control of gambiense sleeping sickness relies predominantly on passive and active screening of people, followed by treatment.

**Methods:**

Mathematical modeling explores the potential of 3 complementary interventions in high- and low-transmission settings.

**Results:**

Intervention strategies that included vector control are predicted to halt transmission most quickly. Targeted active screening, with better and more focused coverage, and enhanced passive surveillance, with improved access to diagnosis and treatment, are both estimated to avert many new infections but, when used alone, are unlikely to halt transmission before 2030 in high-risk settings.

**Conclusions:**

There was general model consensus in the ranking of the 3 complementary interventions studied, although with discrepancies between the quantitative predictions due to differing epidemiological assumptions within the models. While these predictions provide generic insights into improving control, the most effective strategy in any situation depends on the specific epidemiology in the region and the associated costs.

Gambiense human African trypanosomiasis (HAT) is a parasitic disease caused by *Trypanosoma brucei gambiense* and is transmitted by tsetse. Infection occurs in 2 stages, with second-stage disease almost always fatal without treatment. Although numbers of HAT cases have declined since their historic highs in the 1940s and late 1990s, HAT remains a significant health burden in multiple foci, particularly in the Democratic Republic of the Congo (DRC).

Gambiese HAT is targeted for “elimination as a public health problem” by 2020, which is defined as a 90% reduction in areas reporting >1 case in 10000 compared to 2000–2004, and <2000 annually reported cases globally [1]. In addition to this goal, there is a global 2030 elimination goal (zero transmission). In 2016 there were <2200 reported cases of gambiense HAT compared to >25000 in 2000 (http://apps.who.int/neglected_diseases/ntddata/hat/hat.html). The related infection, rhodesiense HAT, constituted just 3% of human infections in 2014 [1], but the zoonotic nature of transmission is considered to make interruption challenging and consequently it is not currently targeted for elimination, nor examined further in this study.

Current gambiense HAT treatments are specific to disease stage and have significant side effects. Therefore, at-risk populations must be screened and the presence of the parasite confirmed before being treated. Adherence to screening programs is known to be highly heterogeneous within targeted communities. Both anecdotal observations [2] and model inference [3, 4] suggest that particular demographic groups, such as those working away from the village during screening days, are both at higher risk of infection and less likely to be screened, potentially forming a human reservoir of infection, reducing the impact of active screening campaigns [5, 6].

Other forms of reservoir may also exist. While multiple animal species have been found to harbor trypanosomes, their role within the transmission cycle remains unclear [4, 7, 8]. Recent studies have also suggested that a nonnegligible proportion of infected individuals may tolerate infection without developing symptoms or detectable levels of parasites in their blood [9–11]. These asymptomatic individuals could be a plausible driver of the persistence and reemergence of HAT in low-prevalence foci where an animal reservoir is unlikely.

With these multiple complexities affecting the impact of interventions, there are open questions about whether the 2020 and 2030 targets can be reached using existing strategies. Modeling studies have suggested that, in many areas, current control measures using standard medical-only strategies lead to a sustained reduction in transmission [4, 12]; however, to reach the 2030 goal of zero transmission, additional interventions will likely be needed, particularly in areas of persistent transmission [5, 12].

This article explores 3 additional interventions that are currently available but have not typically been integrated into HAT strategies: vector control, enhanced passive surveillance, and targeted active screening. We first highlight results from previous modeling studies on the effectiveness of these strategies and then use 4 state-of-the-art models to explore the potential of these strategies in high- and low-transmission settings.

## Insights From Previous Modeling Studies

Two medical interventions are core components deployed in many HAT-affected areas:

1) Passive surveillance: Self-presentation by HAT-infected people to medical facilities. Infections are generally detected during stage 2 disease—when symptoms are more severe and specific to HAT.
2) Active screening: Mobile teams screen and diagnose HAT patients in at-risk locations. Once detected, patients travel to medical centers for treatment. In this study it is assumed that 30% of the population is screened each year. Models including population heterogeneity in exposure to tsetse assume that only low-risk people are screened under this intervention.

**Figure 1.**
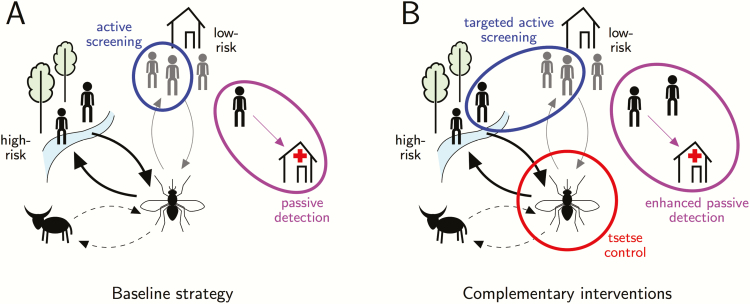
Schematic of the human African trypanosomiasis transmission cycle, showing baseline medical interventions (*A*) and complementary interventions using currently available tools considered in this study (*B*) (adapted from [30]). *A*, Baseline interventions: passive detection of infected individuals via medical facilities (purple), and active screening (blue). Models with high- and low-risk people assume that high-risk people receive more bites from tsetse (thicker arrow) and only low-risk people are actively screened. *B*, Additional interventions: (1) Tsetse control (red) directly impacts all transmissions; (2) enhanced passive surveillance improves access and detection at health facilities (purple); (3) targeted active screening improves uptake of active screening campaigns and high-risk people are assumed to participate equally to low-risk people (blue). In some model variants, animals act as a sink to tsetse bites but do not contribute to transmission (dashed arrow).

In addition there are 3 main interventions (Figure 1) that are the focus of this comparative analysis and have been modeled in previous studies:

3) Enhanced passive surveillance: People self-present; however, the time to detection can be reduced by improved access to HAT diagnostics. The present study assumes that this doubles the detection rate, although realistic increases have not yet been quantified.
4) Targeted active screening: This improves active screening by increasing the population coverage from 30% to 60% annually. Mobile teams are also assumed to target both high- and low-risk people equally. This should not be confused with reactive screening (not modeled here), which refers to active screening in a given location following detection of a case by passive screening.
5) Vector control: This is assumed to reduce the tsetse population by 60% after 1 year by using tiny targets to attract and kill flies.

First, insights from previous modeling studies on these additional interventions are reviewed, before performing new analyses comparing these strategies using different modeling frameworks.

## Vector Control

Vector control has considerable potential in reducing transmission but is not currently a main strategy for HAT elimination. Although vector control does not reduce the disease burden in humans already infected, it reduces biting on all hosts and can complement medical interventions. Mathematical modeling of various vector-borne diseases shows that vector targeting can be highly effective in reducing transmission [13–15] and does not need to completely eliminate the vector to interrupt transmission.

For HAT, tsetse populations have been reduced by at least 80% in various scenarios using “tiny targets” [3, 16, 17]. These are typically more cost-effective than other tsetse control methods such as large targets, aerial spraying, and sterile insect release [18, 19] and so modeling studies have often focused on this control. In the Boffa focus of Guinea, the introduction of tiny targets in 2012 resulted in a reduction of both tsetse density (80%) and disease prevalence in targeted vs nontargeted areas [16]. Modeling indicated that expanding vector control across Boffa would be very effective for locally achieving elimination goals even with a reduced frequency of active screening, including interruptions that occurred during the Ebola outbreak in 2014–2015 [8]. In the Mandoul focus of Chad, tiny targets were introduced in 2014, leading to a substantial decline in tsetse abundance (99%) and reductions in cases detected by both active and passive screening. A model of HAT transmission indicated that 70% of the case reduction between 2013 and 2015 may be attributable to vector control [3].

The country with the highest HAT burden, DRC, has not yet incorporated vector control into its national strategy. A field trial in Yasa-Bonga health zone (former Bandundu province) has indicated that tiny targets have the potential to reduce fly populations by approximately 80% in this region (S. Torr, personal communication). While medical interventions appear to have greatly impacted transmission throughout the region, in some areas, such as Kwilu and former Equateur province in DRC, modeling predicts that this strategy alone is not sufficient to meet the full elimination target by 2030 [4, 12]. In these areas, projections from 2 different families of model suggest that even moderately effective vector control (60% vector reduction) would be highly effective at reducing transmission, and could help these regions to achieve elimination by 2030 [5, 12].

Dynamic transmission models have been integrated into economic frameworks to evaluate the cost-effectiveness of vector control. They suggest that, in high-transmission settings, elimination was likely and cost-effective only when vector control was integrated to the control strategy. However, in low-transmission settings, vector control was not found to be cost-effective [6, 20].

## Enhanced Passive Surveillance

Passive surveillance, a core component of HAT strategy, enables the diagnosis and treatment of symptomatic patients, reducing disease burden and mortality. In DRC, approximately 50% of cases are detected passively [21]. Correctly identifying and treating HAT cases remains complex due to, among other things, the nonspecific presentation of symptoms in early infection, lack of awareness by healthcare workers and patients [22], and scarcity of diagnostics in local health facilities [23]. Recently, in foci in Uganda, Chad, Côte d’Ivoire, Guinea, Nigeria, DRC, and Angola, efforts have been made to improve access to diagnosis and treatment by equipping health facilities with both rapid diagnosis tests (RDTs) and confirmatory diagnostics (https://www.finddx.org/ntd/hatprojects/implementation-of-hat-diagnostics/), with the aim of improving population coverage and time to diagnosis.

Modeling studies have evaluated the potential impact of an enhanced passive strategy. One study simulated enhanced passive surveillance by increasing the detection rate 2-fold to mimic the new RDT strategy that was implemented in Chad in 2015 [3]. The results suggest that RDTs led to an increase in case detection and reporting in 2015, although in subsequent years, the associated reduction in transmission leads to the same or fewer cases being reported.

## Targeted Active Screening

Infectious diseases generally have highly heterogeneous risk, and increases in the efficacy of control can be achieved by targeting the higher-risk groups [24]. For HAT, there is evidence of persistent underrepresentation of the same groups at screening [2], suggesting that current active screening methods are likely suboptimal and may often not include those that are most at risk. Therefore, the efficacy of active screening campaigns may be improved by reducing systematic nonparticipation, potentially by mini teams who screen from house-to-house rather than in the village center. A door-to-door mobile screening strategy in Côte d’Ivoire was found to detect significantly more HAT cases than standard active screening [11]. Previous modeling studies have shown that targeted screening, resulting in increased coverage of high-risk groups, could lead to a greater chance of HAT elimination in some high-endemicity settings [5, 6].

One key element often missing from model-based predictions and analysis is sensitivity analysis to model formulation, and the different disease-specific heterogeneities that are included. Previous studies have highlighted how a comparison of different model predictions can be highly informative, especially in terms of developing policy-relevant consensus [12, 25]. Here we bring together 4 different models and investigate areas of agreement and uncertainty.

## METHODS

Four state-of-the-art mathematical models, labeled models I, S, W, and Y (see details in the Supplementary Materials) are used to illustrate the impact of vector control, enhanced passive surveillance, and targeted active screening on transmission in high- and low-risk settings. Baseline strategies, consisting of both passive surveillance and active screening by mobile teams covering 30% of the population each year, were assumed to occur in 2000–2017, followed by the strategies in Table 1 for 2018–2030. These additional strategies are compared by simulating the reduction in new infections (2018–2030) as well as the probability of local elimination, defined here as <1 new infection per million individuals per year.

**Table 1.** Strategies Under Consideration (2018–2030)

Interventions	Strategy Name			
	Baseline (2000–2017)	Vector Control	Enhanced Passive Surveillance	Targeted Active Screening
Basic passive detection rate	Y	Y		Y
Passive detection rate doubled			Y	
30% active screening	Y	Y	Y	
60% active screening (with equal coverage of low- and high-risk people)				Y
Tiny targets with 60% tsetse reduction after 1 year		Y		

## RESULTS

**Figure 2.**
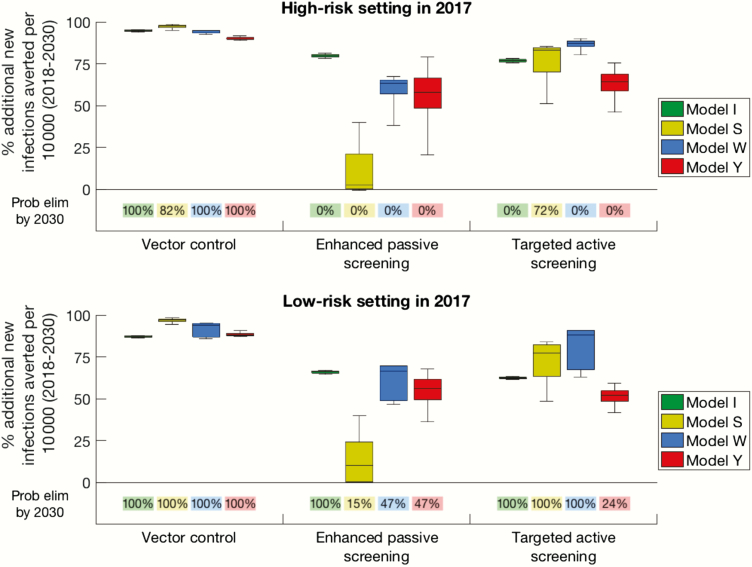
Results of model simulations in high-risk (*A*) and low-risk (*B*) settings for 3 strategies including either vector control (60% reduction in tsetse population), enhanced passive surveillance (double the detection rate), and targeted active screening (double the coverage, including screening high-risk groups). Percentages below boxes denote the probability of a strategy leading to elimination (<1 transmission per 1000000 population) by 2030 for each model. This figure, which displays percentages rather than absolute numbers of infections averted, takes into account the variability between models in the baseline number of new infections expected.

The models agreed that the baseline strategy would not be sufficient to meet elimination by 2030 in all high-risk and many low-risk settings, suggesting that pursuing improved intervention strategies would be beneficial (Supplementary Table 2). All 4 models agreed that a sustained 60% tsetse reduction can greatly affect transmission of HAT and would very likely lead to the 2030 elimination target in both high- and low-risk settings (Figure 2 and Supplementary Figures –7).

In the absence of additional vector control, doubling the passive case detection rate may lead to elimination in low-risk areas, but not in high-risk settings (Figure 2 and Supplementary Figures 5–7). However, there was considerable variability between model predictions (Figure 2 and Supplementary Figures 5–7), caused by differences in model structure (eg, whether the population is partitioned into high- and low-risk groups), model assumptions (eg, whether stage 2 disease is infectious) and baseline parameterization (Supplementary Table 1). The 2-fold increase was chosen in this study as an example; however, it is currently unclear what relative increase may be possible. In some regions, the number of health facilities with HAT diagnostics has recently increased substantially (37-fold in Uganda and 18-fold in South Sudan [26, 27]), although the resulting increase in detection rate remains unknown.

Improvements in the number (and the targeting in models S and W only) of participants screened worked well in low-risk settings, leading to elimination by 2030 (Figure 2). Elimination was not predicted in high-risk settings, except by model S, despite the strategy averting a large number of new transmissions in many instances. Models S and W were particularly optimistic about a targeted active screening strategy due to their estimated importance of transmission from high-risk groups.

### Model Comparison

For all models, vector control always averted more infections than the other strategies considered. This result echoes other vector-borne disease modeling [28], where targeting vectors has a nonlinear impact. The 4 models predicted that local elimination would very likely be reached by 2030 if vector control was continuously implemented from 2018, even with moderate efficacy (Figure 2 and Supplementary Figure 5).

There was discrepancy between models over the next best strategy, reflecting different model assumptions and differences in underlying parameters: Models S and W concluded that targeted active screening always averts more transmissions than enhanced passive surveillance, whereas model I found the converse; for model Y the prediction intervals overlap. None of the models found it possible to achieve local elimination by 2030 using enhanced passive surveillance in high-risk settings and only model S found it possible using targeted screening, although these interventions might be sufficient in some low-risk settings. It is noted that the success in local elimination depends on how this concept is defined; given that the models are all deterministic, local elimination was defined as <1 transmission event per million individuals per year. For a weaker definition (<1 transmission event per 100 000 individuals), all models had some low-risk simulations that resulted in elimination for the baseline strategy alone (Supplementary Table 2). True elimination can only be assessed through stochastic simulations that recognize the individual nature of the populations and the risk of external imports of infection.

Both improved medical strategies considered for this study are likely to depend on the behavior of the population (eg, whether high-risk people are actively screened and the utilization of RDTs in health facilities). Population-level heterogeneities are incorporated in different ways in the different models, which partly explains the varying outcomes for the 2 medical interventions.

## DISCUSSION

### Future Strategies and Tools

As HAT prevalence decreases, active screening will likely shift toward a reinforced passive surveillance system due to limited resources and greater cost-effectiveness. In such situations, a reactive screening strategy would be an obvious complement and has already been used in parts of Uganda and DRC. This strategy could provide an economical way to monitor and treat HAT in areas with little ongoing transmission. The optimal timing of a switch from active to reactive screening is unclear, as it is influenced by the strength of the underlying passive surveillance system, the risk of imported infection, the presence of other control measures (such as vector control), and the relative costs of treatment and screening. Therefore it is paramount to monitor availability and uptake of the passive system and to develop measures to identify where improvements could be made. As we move toward the endgame of HAT elimination, passive surveillance will be a key component of how progress is measured.

Current treatment requires that a lumbar puncture be performed on all patients and that those in stage 2 disease be admitted to hospital, which creates barriers for treatment and compliance [2]. Orally administered drugs, currently in the pipeline (https://www.dndi.org/diseases-projects/portfolio/), could drastically change treatment protocols and alter current strategies or even the paradigm for HAT strategy within the next few years.

### Modeling Assumptions

Models were calibrated to be representative of regions of high or low transmission, highlighting indicative benefits of intensified strategies rather than matching any particular setting. Future model fitting to foci-specific data could help elucidate the potential benefits of these and other interventions in a particular setting. Furthermore, modeling improvements could help to understand the effects of land use changes (eg, human behavior, human migration, or tsetse habitat destruction) on disease dynamics.

For low-transmission settings in particular, time to local elimination and the probability of elimination may be influenced by chance events. Stochastic models will help to refine these estimates by explicitly accounting for the likelihood of disease elimination through natural failure of transmission events. These models could also explore chance and timescales of possible recrudescence of disease triggered by lack or loss of interventions in conflict areas, importations of new infection to locally eliminated foci (eg, by displaced populations), or premature cessation of an elimination program.

The present study focuses on the impact of interventions on transmission and not on reported cases. Although implementing vector control immediately results in fewer new transmissions, it may take several years to observe a reduction in reported cases due to the long timescale of HAT infection; for strategies that improve the detection of cases, there may be a brief rise in reported cases before this number decreases.

In this study a conservative tsetse reduction was used, lower than observed reductions in Guinea (80% reduction) [16], Uganda (>90%) [17], Chad (99%) [3], and DRC (~80%; S. Torr, personal communication). Geographic specificities will impact the ease and frequency of target deployment and tsetse population response to the intervention.

Likewise, the impact of enhanced passive surveillance and targeted active screening has not yet been fitted to data. It is nontrivial to assess the relationship between the number/location of health facilities with diagnostics/treatment and the improvement in time to detection. Hence, predictions can be improved by analyzing the observed impact on case reporting in regions where these interventions have previously been conducted.

### Operational and Financial Feasibility

The model simulations considered constant levels of intervention effectiveness for the different strategies. In reality, this will vary geographically and temporally, and will depend on factors outside the control of the health system, such as infrastructure and political stability.

The results shown here describe the impact of different interventions in averting infections but do not consider the costs or feasibility associated with achieving the assumed coverage level of those interventions. For example, the cost of implementing tiny targets has been estimated at US$85.4 per km^2^ per year [18], while in Uganda additional health facilities are estimated to cost about US$425 per year [29]. A tailored, focus-specific modeling approach is likely needed to best capture the local geography, operational feasibility, and associated costs of such strategies.

## CONCLUSIONS

All models agreed that vector control would consistently avert most infections and likely lead to elimination by 2030 in all considered scenarios, whereas there was some discrepancy over the next best strategy mainly driven by uncertainty in key epidemiological processes, such as numbers and infectivity of different infected individuals. Targeted active screening and enhanced passive surveillance are both predicted to be very effective, particularly in low-risk settings, but unlikely to lead to elimination in high-risk settings despite averting many additional transmission events.

Models need more detailed, up-to-date data to parameterize strategies in different settings to provide the best guidance, as the uncertainties in the predictions demonstrate. As new interventions become available and are deployed, it will be essential for modelers, field researchers, and the HAT community to work together to better understand the effectiveness of interventions in reducing transmission and achieving elimination.

## Supplementary Data

Supplementary materials are available at *Clinical Infectious Diseases* online. Consisting of data provided by the authors to benefit the reader, the posted materials are not copyedited and are the sole responsibility of the authors, so questions or comments should be addressed to the corresponding author.

Supplementary Material

## Notes

***Acknowledgments.*** The authors thank the numerous collaborators whose expertise in HAT control has improved the clarity and precision in this modeling article, including Prof Philippe Büscher, Dr Epco Hasker, and Prof Steve Torr. Calculations for the S model were performed at the sciCORE (http://scicore.unibas.ch/) scientific computing core facility at the University of Basel.

***Disclaimer.*** The views, opinions, assumptions, or any other information set out in this article are solely those of the authors and should not be attributed to the funders or any person connected with the funders.

***Financial support.*** This work was supported by the Bill & Melinda Gates Foundation (BMGF) in partnership with the Task Force for Global Health through the Neglected Tropical Disease (NTD) Modelling Consortium (award number OPP1053230 to K. A., A. G., T. D. H., M. J. K., M. N. B., A. P., K. S. R.), BMGF (award number OPP1156227 to N. C., S. C., A. G., M. J. K., T. D. H., M. N. B.), the Children’s Investment Fund Foundation (UK), and Novartis Foundation. C. P. and C. A. B. are supported by BMGF through the Global Good Fund. J. N. is supported by funding from BMGF (award numbers OPP1154033 and OPP1033712), the Swiss Agency for Development and Cooperation (award number 81050188), the UK AID Department for International Development (award number 81050188), and the German Federal Ministry of Education and Research.

***Supplement sponsorship.***This article appears as part of the supplement “Reaching the 2020 Goals for Nine Neglected Tropical Diseases,” sponsored by the NTD Modelling Consortium.

***Potential conflicts of interest.*** All authors: No reported conflicts of interest. All authors have submitted the ICMJE Form for Disclosure of Potential Conflicts of Interest. Conflicts that the editors consider relevant to the content of the manuscript have been disclosed.
