# Supplementary material for: Assessing Strategies Against Gambiense Sleeping Sickness Through Mathematical Modeling
Source: Clin Infect Dis. 2018 Jun 1;66(Suppl 4):S286–92. doi: 10.1093/cid/ciy018 (PMC5982708; doi:10.1093/cid/ciy018)
Supplement: Supplementary Material [file ciy018_suppl_supplementary_material.zip › AssessingHATStrategySI_040118.pdf]

Supplementary Information

Assessing strategies against gambiense sleeping sickness through mathematical modelling

Kat S Rock*[1,2], Martial L Ndeffo-Mbah[3], Soledad Castaño[4,5], Cody Palmer[6], Abhishek Pandey[3], Katherine E Atkins[7], Joseph M Ndung'u[8], T Déirdre Hollingsworth[1,2,9], Alison Galvani[3], Caitlin Bever[6], Nakul Chitnis[4,5], Matt J Keeling[1,2,9].

*Corresponding author

Affiliations

1 Zeeman Institute for Systems Biology & Infectious Disease Research (SBIDER), The University of Warwick, Coventry, CV4 7AL, UK
2 School of Life Sciences, The University of Warwick, Coventry, CV4 7AL, UK
3 Yale School of Public Health, Yale University, New Haven, CT, 06510, USA
4 Department of Epidemiology and Public Health, Swiss Tropical and Public Health Institute, Socinstrasse 57, Postfach, 4002 Basel, Switzerland
5 University of Basel, Petersplatz 1, Postfach, 4001 Basel, Switzerland
6 The Institute of Disease Modeling, 3150 139th Ave SE, Bellevue, WA 98033, USA
7 Department of Infectious Disease Epidemiology, Faculty of Epidemiology and Population Health, London School of Hygiene and Tropical Medicine, London, WC1E 7HT, UK
8 Foundation for Innovative New Diagnostics, Geneva, Switzerland.
9 Mathematics Institute, The University of Warwick, Coventry, CV4 7AL, UK

In this document we present three main additional items. We first provide more information on the three additional strategies studied within the main paper; we provide a comprehensive overview of the four models used; and finally we present our findings in alternative formats to highlight differences in absolute numbers and temporal dynamics.

1. DEFINING THE THREE INTENSIFIED STRATEGIES IN HIGH- AND LOW-RISK SETTINGS

The 2020 goals are global measures, yet infection and disease transmission are inherently local processes. This means that without screening human populations across all areas of Africa which are currently and historically HAT-affected it is difficult to ascertain if the goals have been reached. For this reason, models often focus on a simpler measure of the predicted success of control measures -- the number of new infections averted over a given time span. The results presented here show the total number of additional new infections averted between 2018 and 2030 under each complementary strategy compared to a baseline strategy of annual active case screening and typical passive surveillance.

In order to examine the impact of these three tools, each of the models (Models I, S, W and Y) were used to project forwarded (a) a baseline, medical-only strategy which included both active detection (screening 30% of the population each year) and basic passive surveillance, (b) a vector-control strategy of moderate efficacy using tiny targets in addition to the baseline medical intervention, (c) an enhanced passive surveillance strategy using diagnostics including RDTs to double the detection rate in addition to 30% annual active screening, and (d) a targeted active screening intervention which doubles the coverage in addition to basic passive surveillance. Table 1 outlines each strategy which is assumed to begin in 2018, following 18 years of the baseline strategy (2000–2017).

"High-risk" settings, according to WHO definition, have between 100 and 1000 annually reported cases on average across a given time period (e.g. 5 or 10 years) per 100,000 people, whereas "low-risk" settings have between 1 and 10 cases for the same population and have already met the "elimination as a public health problem" locally, yet still have transmission [1, 2]. Since the number of reported cases in a region is inherently reliant on the amount of active screening and quality of the passive surveillance system, the present study uses related but different thresholds of between 500-600 annual new infections per 100,000 people for "high-risk", and 5-6 new infections per 100,000 for "low-risk". These narrower ranges factor in even a relatively high level of underreporting and the models matched to these transmission incidences are designed to reflect generic settings which would be classified as high- and low-risk respectively in 2017.

As all the models are deterministic, a cut-off threshold for elimination needed to be selected. As the WHO defines a "marginal-risk" setting as <1 annually reported case per 1,000,000, the threshold of <1 new transmission per 1,000,000 per year was chosen as the elimination threshold used in the main text. A least stringent threshold of <1 case per 100,000 was also examined in Table S3 to see how this would impact the predicted probability of elimination under different strategies.

2. DESCRIPTION OF FOUR MODELS

i) Model I (IDM)

The IDM HAT model is a compartmental model for the spread of the disease that aims to capture the most prominent and well known aspects of the disease. Susceptible individuals are infected by interacting with infected flies in a frequency-dependent way. This interaction is also governed by a biting rate and a vector competency term. Once infected, individuals move into the first stage of the disease, and are picked up throughout the year by passive surveillance at a given rate. If not identified in surveillance, the individuals proceed to the second stage of the disease, where they have further opportunities to be detected through surveillance (see Figure S1). Active surveillance is modeled through a discrete process where, once a year, a certain percentage of infected individuals are placed into the recovered compartment. If not detected through active screening or passive surveillance, it

is assumed that an infected individual will eventually die. Infected individuals picked up through surveillance are placed in a recovery compartment where they cannot be infected. This lasts for the duration of treatment, and thereafter individuals proceed back into the susceptible population. The flies move from a susceptible population to a exposed compartment where the await the completion of the trypanosomes life cycle in their midgut. This transition is governed by biting rates and host competencies for infectious individuals, and there is no assumption of equality or inequality for the two host stages. The flies then move into the infected class where they can pass the parasite onto hosts.

Model Samples. To fit the model, data from WHO HAT Atlas [1, 3] was used for a collection of HAT-endemic foci in the DRC.
Given the relatively low dimensionality (n=5) of this model's parameter space, parameter uncertainty was explored using waves of Latin Hypercube Sampling where parameter values that generated non-conformable disease profiles with the data were excluded. With each wave the parameter space was reduced and sampling was repeated until stabilization occured. With these parameters ranges in hand, we scaled $\square_0$ to generate desired incidence for the high- and low- risk settings and generated 1000 parameter sets for each.

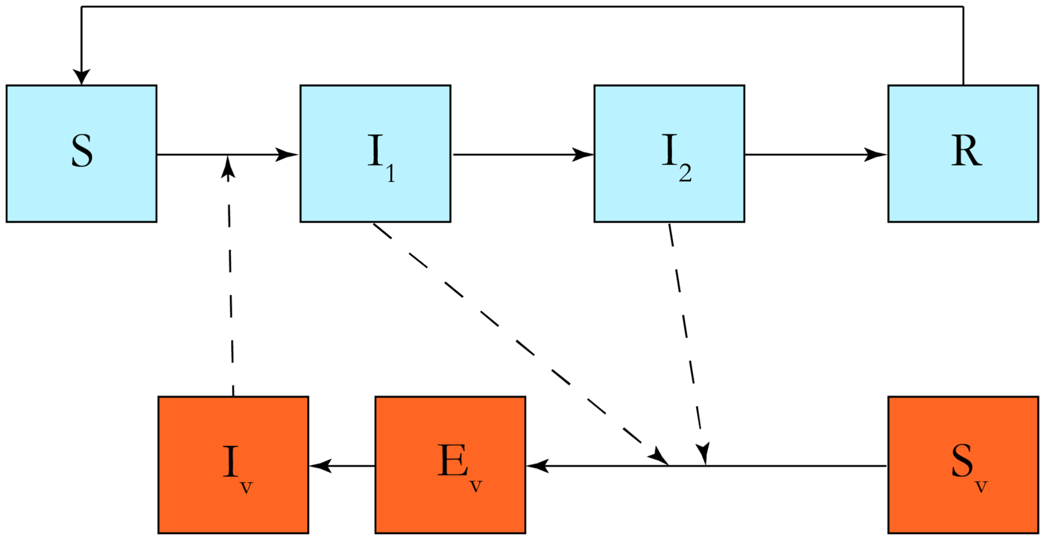

Figure S1. Schematic for the IDM model, showing the host and vector population structures and the interactions that drive the spread of the disease.

Intervention impact. The three additional strategies are implemented within the basic framework as follows:
Vector control via tiny targets was implemented by decreasing the vector population 60% within one year.
Enhanced passive surveillance was simulated by increasing the rate of transfer of stage 1 individuals into the recovered class by a factor of 2. This was done preferentially for stage 1 individuals, under the assumption that introduction of RDTs to health facilities allows health

workers to more accurately diagnose the first stage of the disease, while the unique symptoms of the stage 2 remain prominent.

Targeted active screening was implemented by increasing the percentage of individuals captured by active screening in both stages of HAT two-fold.

ii) Model S (Swiss TPH)

The Swiss TPH model is a deterministic compartmental model consisting of a system of coupled ordinary differential equations. The model schematic is shown in Figure S2. Two separate sets of populations of tsetse flies and non-human animals live in the village and in the high transmission area. Tsetse flies have an intrinsic preference for biting humans or animals. In the model variant considered here, animals form a sink for tsetse bites but do not contribute to transmission.

Humans are divided into two groups: a low risk group that remains in the village; and a high risk group that commutes to an area with potentially higher transmission (such as river banks or plantations). The model assumes that stage 1 people in the high-risk group are not detected by either passive or active surveillance. Passive surveillance is modelled with a continuous treatment rate, [illegible]$_{ps}$, while active screening recruits infected people for treatment only in the last month of each year.

In both active and passive surveillance, treated patients do not contribute to transmission during their recovery time, after which they lose immunity, thus returning to the susceptible population. Further details on the model can be found in [4].

Model Samples. Uniform priors were used for all parameters. The ranges explored correspond to those described in Table 2 of [4], except for *N2/N1*, the ratio of humans in the high-exposure group to low exposure; and for r_3, the rate at which treated humans return to the susceptible class. The former was explored in the interval (0.1–1) to avoid unrealistic outputs, while the range considered for r_3 allowed treated individuals to remain immune for between 3 and 8 months after receiving treatment before returning to the susceptible state. The daily removal rate of infected humans due to treatment included r_{ps}, that represents continuous passive surveillance and was explored in the interval (3 x 10^{-4} days^{-1}, 1 x 10^{-3} days^{-1}); and r_{as}, which accounts for a pulsed (1 month each year) active surveillance (see [4] for more details). The infectivity of animals to humans was set to zero so that animals did not serve as reservoirs. Bayesian sampling-resampling was used to select 1000 sets of parameter values that led to an assumed equilibrium prevalence of infection in the year 2000 (on-going passive screening only) with corresponding levels of annual incidence in the specified high- and low-risk ranges in 2017 with 30% active screening.

Intervention impact. The three additional strategies are implemented within the basic framework as follows:

Vector control via tiny targets was simulated by increasing the vector mortality rate so that equilibrium vector population size was reduced by 60% after one year.

Enhanced passive surveillance was simulated by doubling the rate of transfer of individuals from stages 1 (low-risk group only) and 2 to the treatment class..

Targeted active screening was simulated by setting the rate of transfer of all infected humans (low risk and high risk, as well and stage 1 and stage 2) to the treatment class at a value such that 60% of the population was screened after 1 year.

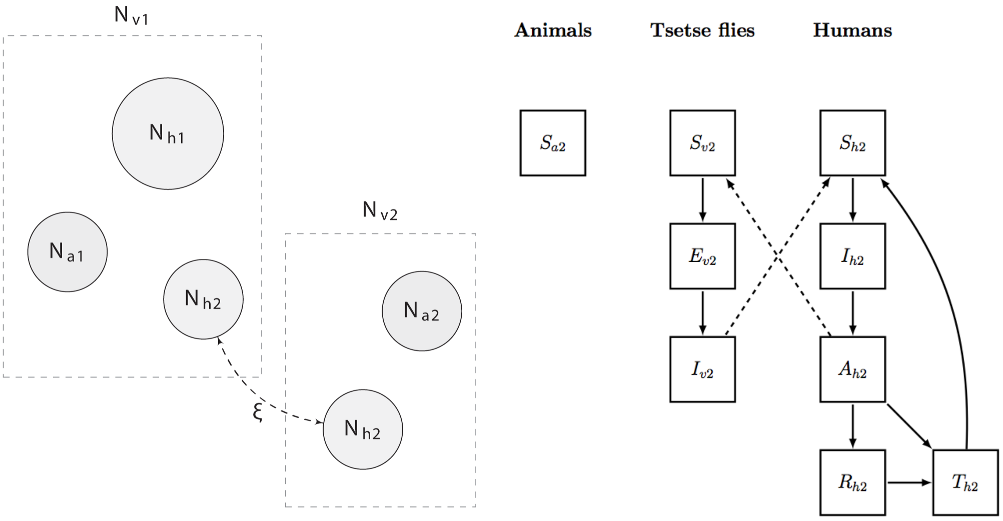

Figure S2. Schematic of Swiss TPH model showing the (A) population structure of low and high-risk individuals and (B) the infectious stages in humans, tsetse flies, and non-human animal hosts. The figure is modified from [4].

iii) Model W (Warwick)

The Warwick HAT model (Model W) is a compartmental ODE model which describes the disease dynamics of human and tsetse populations (see Figure S3). Humans are considered to either be low- or high-risk of exposure to tsetse, with the biting pressure on high-risk being greater. Tsetse are also assumed to feed on other non-reservoir animals which cannot confer infection.

Upon infection from tsetse, humans past through a brief incubation period ([illegible]), and then enter stage 1 disease ([illegible]). During this stage, there is only a small passive detection rate, [illegible], as individuals are may not be picked up through the standard passive surveillance system. After a period of 1/[illegible] on average, undetected people develop stage 2 disease ([illegible]). The passive detection rate increases to [illegible] for stage 2 infection, which has more specific symptoms. Stage 1 and 2 infected people are assumed to be equality infectious to tsetse. Typical active screening (denoted by [illegible]) is assumed to only recruit people from the low-risk group and has a diagnostic algorithm sensitivity of 91%. Screening frequency is determined by the number of people screened - if the number of people screened is lower than the number of low-risk individuals then screening occurs annually, otherwise more

screenings (up to a maximum of 3) occur. After active or passive diagnosis, patients are treated in hospital ([illegible]) and recover at home become returning to a susceptible status.

Tsetse population dynamics are modelled explicitly including a pupal stage ([illegible]), teneral/unfed adults which are susceptible to infection ([illegible]), non-teneral and uninfected adults which have significantly reduced (95%) susceptibility to infection, tsetse incubating infection ([illegible]) and tsetse with mature salivary gland infections ([illegible]).

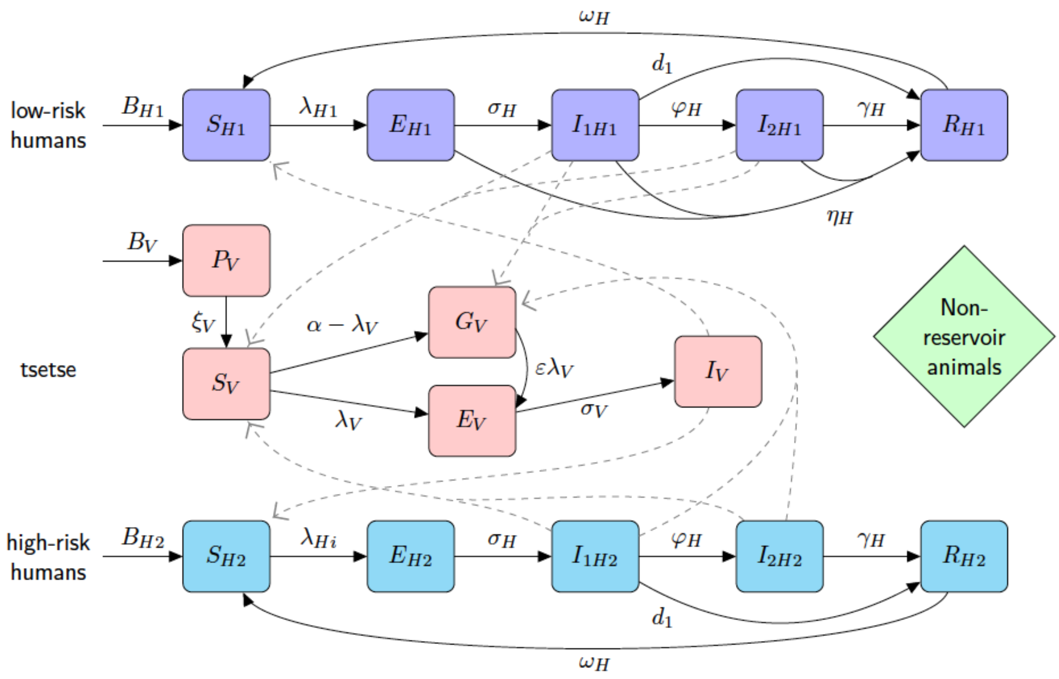

Figure S3. Schematic of Model W. Boxes show compartments representing different types of hosts (blue and purple) and vectors (pink), and disease status and progression for each host/vector type (solid lines). Gray lines show possible routes of transmission between tsetse and humans. This figure is reproduced from [5].

Model samples. Model W takes many of its parameters from the literature (see [5-7]). Following fitting to data from Chad with staged case data, the basic passive detection rate in the present study is assumed to have a mean of 0.000467 days^{-1} compared to the faster stage 2 passive detection rate of 0.006 days^{-1} [8]. This leads to more passive detections in stage 2 than in stage 1. Parameters that were allowed to vary in the present study were: the proportion of low-risk people in the population, the relative risk of high-risk people receiving tsetse bites, and the pre-control tsetse-human density. All of these parameter are considered to vary by setting.

In order to generate model simulations which reflect the parameterisation of endemic areas, posterior distributions from previous analysis of case data from DRC was used [1, 3]. To select parameter space which corresponded with high- and low-risk settings, 1000 posterior parameterisations that lead to either 10–100 or 0.1–1 new transmissions per 10,000 per

year in 2017 were chosen. A baseline active screening of 30% was assumed. These posteriors were calibrated by varying [illegible]$_{0}$ to match uniformly distributed new infection incidences of between 50–60 and 0.5–0.6 respectively.

Intervention impact. The three additional strategies are implemented within the basic framework as follows:
Vector control via tiny targets. There is assumed to be a probability of dying associated with the biting rate, as flies will hit tiny targets and die during the host-seeking stage of the cycle. The probability of hitting a target and dying will change dependant upon how long targets have been deployed for - targets are more effective upon deployment with efficacy waning over time. This additional mortality results in a decrease in the fly population whist targets are deployed but the population can bounce back following target removal or non-replacement. See [7] for further details. Targets were assumed to be deployed biannually in this study and achieve 60% tsetse population reduction after one year.
Enhanced passive surveillance The passive detection rates from stage 1, [illegible]$_{1}$, and stage 2, [illegible], are increased by a factor of 2 to reflect faster time to diagnosis following introduction of RDTs into more health facilities. See [8] for more details.
Targeted active screening. Unlike standard active screening, which is assumed to only recruit low-risk individuals, targeted active screening recruits people from high- and low-risk groups to be screened equally. See [7] for more details. The targeted active screening used here also doubles the number of people screened.

iv) Model Y (Yale)

The Yale model (Model Y) is a deterministic vector-host model for disease transmission between tsetse and human populations. The model implicitly accounts for non-reservoir animals through a biting preference parameter, which ensures that all tsetse bites are not directed to humans. This model was based on our previous models [5, 9].

We assumed that tsetse are susceptible to trypanosome infection only during their first blood meal and only within 24 hours after emergence from pupa to the adult stage. Susceptible adult tsetse become infected after feeding on an infectious human and enter the exposed state where the infection incubates. After incubation, tsetse become infectious for the rest of their life.

Humans may become exposed to infection after being bitten by an infectious tsetse. After the incubation period, a proportion of infected human hosts become symptomatic and enter stage I of the disease (see Figure S4). Infected symptomatic human hosts would progress from stage I to stage II of infection, characterised by the severity of disease symptoms. The remaining proportion of infected human hosts do not become symptomatic and move to an asymptomatic compartment. We assume that these asymptomatic carriers never become symptomatic and recover after an average duration of infection equal to the sum of average durations of stage I and II. We assume that stage I and II are equally infectious, while asymptomatics were assumed to be less infectious. Stage I patients either seek treatment and recover or progress to stage II due to being untreated or due to treatment failure. Stage

II patients either seek treatment and recover or die due to being untreated or due to treatment failure. Successfully treated HAT patients are temporarily immune to reinfection before returning to full susceptibility.

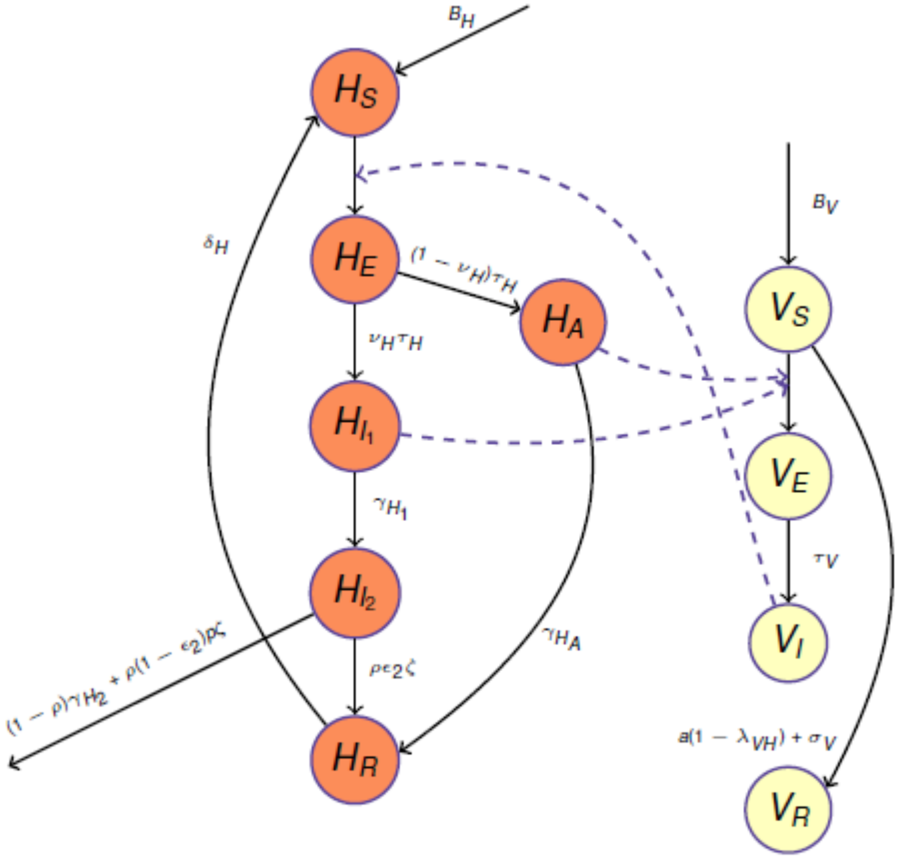

Figure S4. Schematic diagram of the Yale Model showing disease transmission and progression for human and tsetse flies populations. The figure is reproduced from Rock 2017b.

Model Samples. To fit the model to the HAT incidence ranges of interest (0.5–0.6 and 50–60 new cases per 10,000 per year, respectively) in 2017, we assume the system to be at equilibrium in 2000 with only ongoing passive case detection and treatment. Using Bayesian melding approach, the model was fitted to incidence ranges using parameters values described in previous publications as prior distribution [5, 9]. A total of 1000 parameter sets were generated.

Intervention impact. The three additional strategies are implemented within the basic framework as follows:

Vector control via tiny targets. To reduce human - tsetse contact to protect people from infective tsetse bites, we assume that insecticide treated targets were deployed in areas where people live and work. Consistent with previous model calibration work [9], we assumed that for the initial three months of deployment, the targets induced a tsetse maximal kill rate, which decreased linearly to zero in the following three months, and remained at zero until the targets were replaced. Calibration was done to ensure that

biannual deployment of tiny targets reduces tsetse population density by 60% over the first year.
Enhanced passive surveillance. The passive detection rate from stage 1 and stage 2 is increased by a factor of 2 to reflect faster time to diagnosis following introduction of RDTs into more health facilities.
Targeted active screening. As the model does not account for heterogeneity in human health seeking behavior or risk of infection, enhanced active screening was simply modeled as doubling the baseline active screening coverage from 30% to 60%.

Table S1. Overview of models

	Model I	Model S	Model W	Model Y
Deterministic	Y	Y	Y	Y
Partitions population into high / low risk.	N	Y	Y	N
Asymptomatic infection	N	N	N	Y
Animal reservoir	N	N	N	N
Stages assumed to be infectious	1 & 2	stage 1 only	1 & 2	1 & 2
Passive detection at stage 1	Y	Y	Y	Y
Discrete screening events	Y	Y	Y	Y

3. ADDITIONAL RESULTS

The simulation results given in the main paper (Figure 2) can we viewed in multiple ways to highlight different aspects of the problem. Here we give three alternative presentations of our findings, focusing on the probability of local elimination, the number of infections averted and the temporal dynamics following control.

i) Probability of local elimination
Given the models are all deterministic we have to defined local elimination in terms of a simple threshold. In the main paper we consider a threshold of less than one transmission event per million individuals per year. Here we contrast this threshold with a weaker definition of less than one transmission event per hundred thousand individuals per year (Table S2). Here the probabilities given represent the proportion of parameter values (out of all of those that match the high-risk and low-risk definition) that achieve the threshold by 2030.

Table S2. Probability of different strategies achieving elimination according to each model's simulations, using either a threshold of <1 new transmission per 100,000 people, or a threshold of <1 new transmission per 1,000,000 people.

	Setting in 2017	Model	Local elimination probability from simulations			
			Baseline	VC	Enhanced passive	Targeted AS
Threshold (<1 per 100,000 = WHO's "very low risk")	High-risk	IDM	0	1	0.57	0.01
		Swiss TPH	0	1	0	1
		Warwick	0	1	0	0.72
		Yale	0	1	0.04	0.01
	Low-risk	IDM	1	1	1	1
		Swiss TPH	0.34	1	0.43	1
		Warwick	0.46	1	1	1
		Yale	0.63	1	1	1
Threshold (<1 per 1,000,000 = WHO's "marginal risk")	High-risk	IDM	0	1	0	0
		Swiss TPH	0	0.82	0	0.72
		Warwick	0	1	0	0
		Yale	0	1	0	0
	Low-risk	IDM	1	1	1	1
		Swiss TPH	0	1	0.15	1
		Warwick	0	1	0.47	1
		Yale	0	1	0.47	0.24

For the more stringent threshold, only vector control is predicted to lead to elimination in all scenarios investigated. Model I predicts that elimination will be achieved under baseline controls (and hence also with additional strategies) for the low-risk setting. Both Models W and Y predict that in the low-risk setting, elimination may be possible with other additional strategies (Enhanced passive screening and Targeted active screening). Model S suggests that elimination is very likely with targeted active screening and unlikely with enhanced passive detection or the baseline strategy.

For the weaker threshold, the probabilities of elimination (defined as less than one infection event per 100,000 people per year) increase as expected. Both Models W and Y now predict that in the low-risk setting elimination will occur for all additional strategies; while Model S continues to be more pessimistic about the baseline strategy and enhanced passive detection. In a high-risk setting, Models I, S W now predict a reasonable chance of elimination with Targeted active screening, although Model Y continues to predict that elimination will not occur.

ii) Number of Averted Infections

The main paper (Figure 2) focused on the percentage of additional cases averted (2018–2030) relative to the baseline case. While this provides a good comparative measure, it hides fundamental differences between the baseline predictions of the four models. In Figure S5, the raw number of additional new infections averted is shown, which while qualitatively similar to Figure 2, highlights the profound differences between high- and low-risk settings.

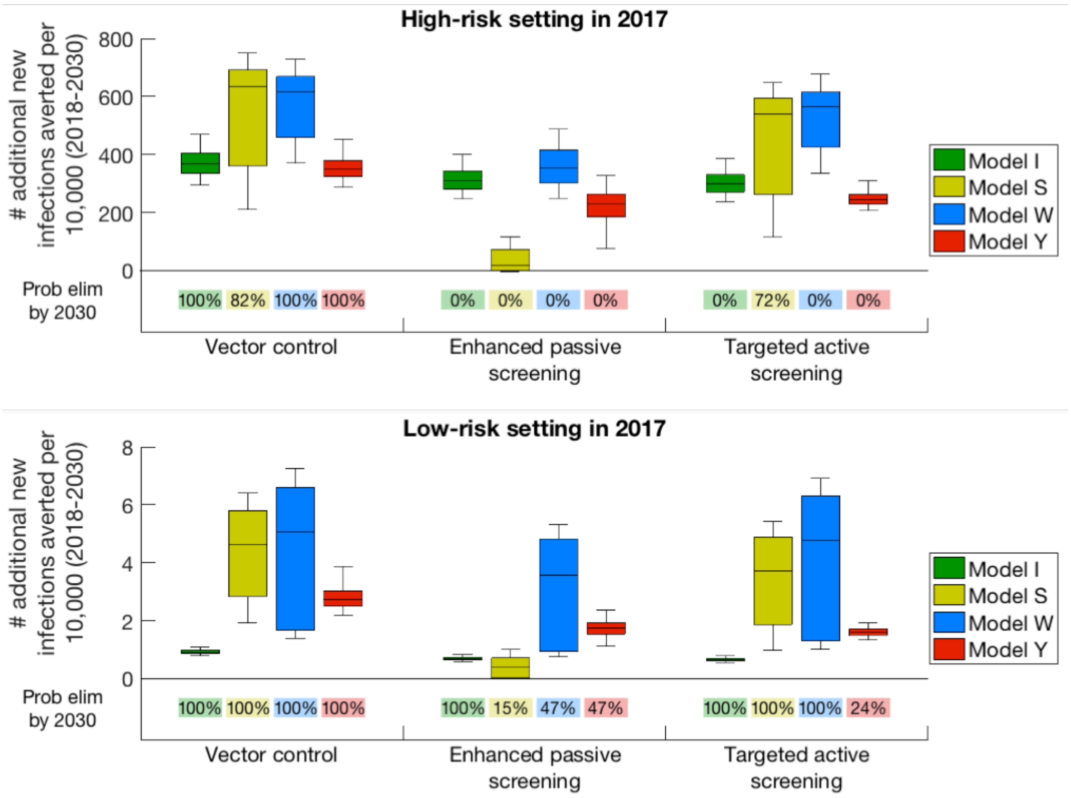

Figure S5. Results of model simulations for three strategies including either vector control (60% reduction in tsetse population), enhanced passive detection (double the detection rate), and targeted active screening (double the coverage, including screening high-risk groups). Boxes show the median, interquartile range of the number of additional new infections averted compared to baseline intervention during 2018–2030, whilst whiskers denote the 95% credible intervals from parameter uncertainty. Percentages below boxes denote the probability of a strategy leading to elimination (<1 transmission per 1,000,000 population) by 2030 for each model.

iii) Temporal evolution of HAT incidence.

Figures S6 and S7 compare the estimated changes in HAT incidence when different strategies are applied in 2018–2030 in both high- and low-risk transmission settings.

In general all models suggest that current strategies (i.e. baseline) will fail to halt HAT transmission by 2030 (using the <1 infection per 1,000,000 threshold), with Model I being an exception for the low-risk settings. The models have quite different baseline prediction intervals (shown in grey), with Models S and W having quite wide ones and Models I and Y having quite tight ones. Model W (and Model S in high-risk settings) had numerous simulations which resulted in little impact of the baseline strategy on new infections, whereas Models I and Y produced substainal decline under this strategy alone.

All models show a rapid and dramatic impact of vector control, with relatively narrow uncertainty. For strategies targeting the infected human population, although there is no agreement among models, enhanced passive surveillance or targeted active screening are expected to outperform the baseline strategy in most of simulations.

The divergence observed in estimations and uncertainties rely on model structure and parameterisation differences. For example, Model S considers only the stage 1 of HAT as infective to tsetse, while the remaining models assume stages 1 and 2 to be infective. This difference, combined with the assumption of only low-risk stage 1 individuals being passively diagnosed, explains the lower impact of enhanced passive surveillance observed. Also, in Model S the targeted active screening takes place the last month of each year. Such delay explains the apparent lack of impact on transmission of the first year observed in Figures S6 and S7 for this intervention. Models S and W both have high-risk non-participatory groups in the population driving infection dynamics prior to a change in strategy and so targeted active screening, which tackles the important high-risk human reservoir, performs well under these models.

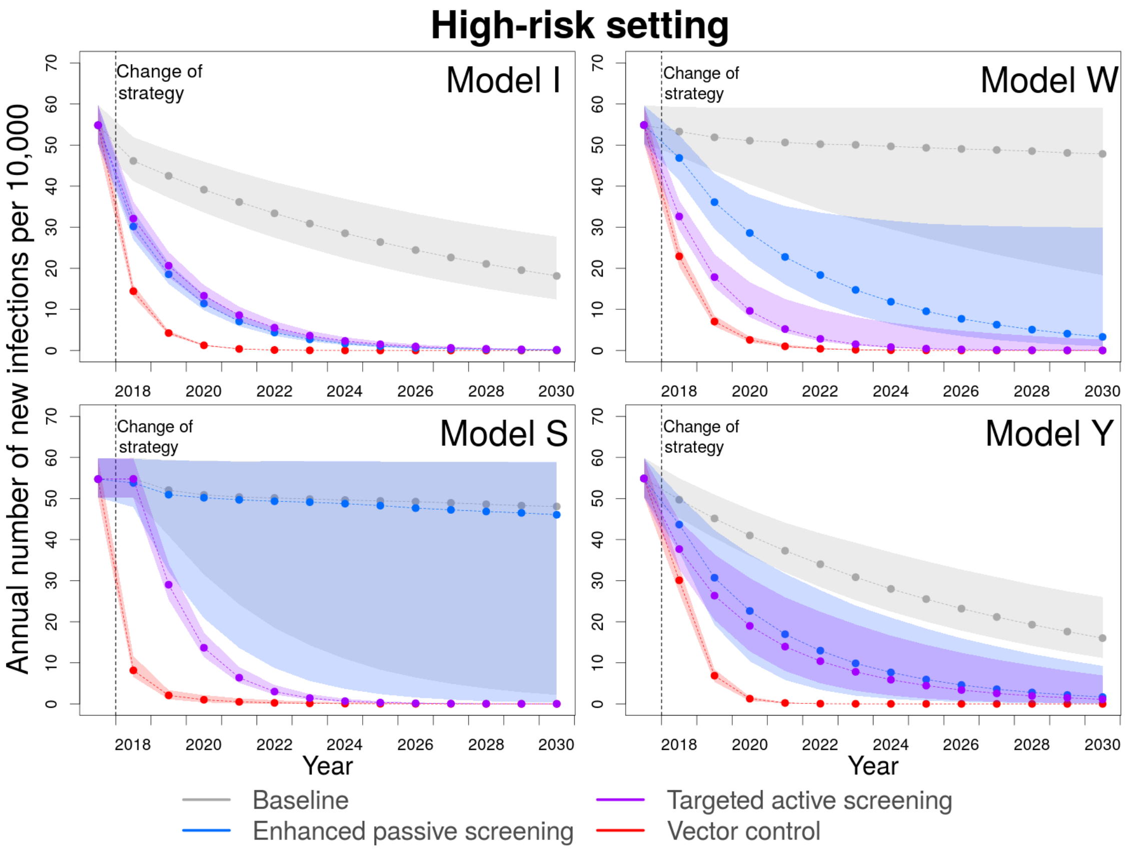

Figure S6. Temporal evolution of HAT incidence in a high-risk transmission setting under different strategies. For each model and strategy considered, the median number of annual new infections across the year under the four strategies is shown as a point and the corresponding 95% CI is shown as the shaded region of the same colour. The dashed vertical line denotes the implementation of new strategies at the start of 2018.

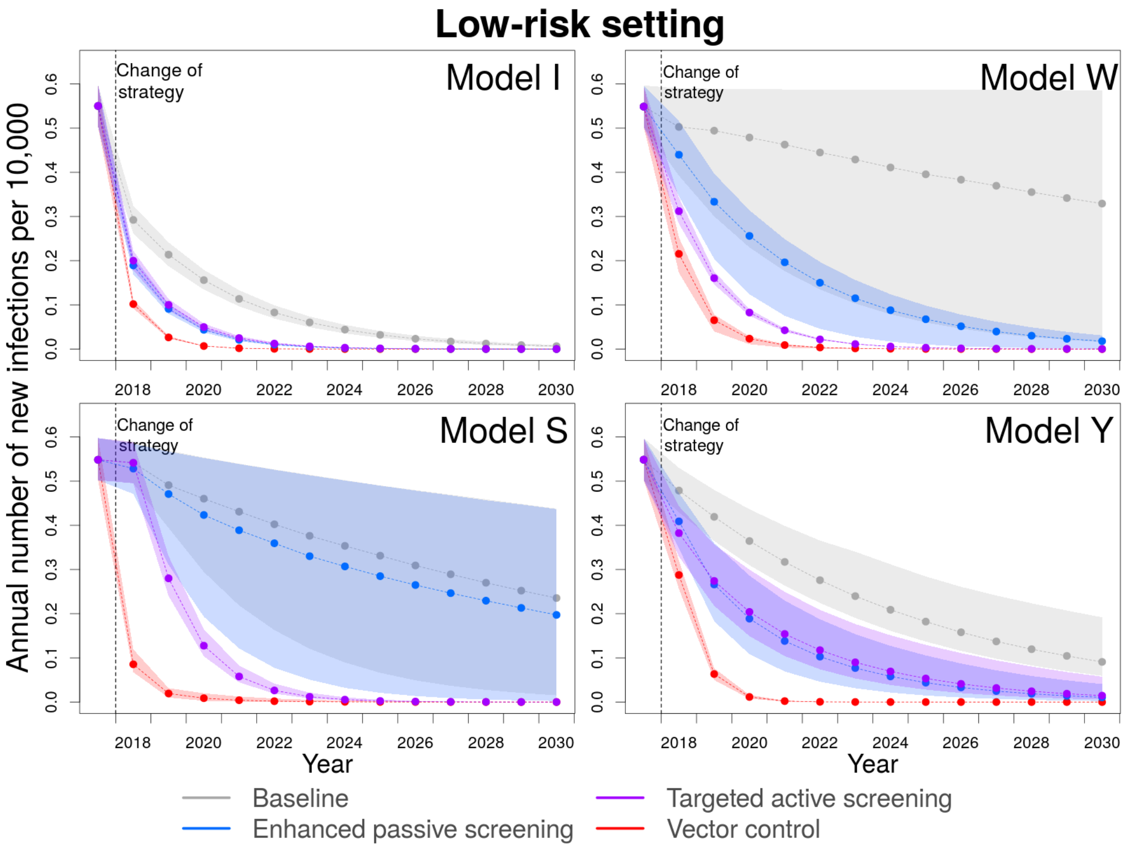

Figure S7. Temporal evolution of HAT incidence in a low-risk transmission setting under different strategies. For each model and strategy considered, the median number of annual new infections across the year under the four strategies is shown as a point and the corresponding 95% CI is shown as the shaded region of the same colour. The dashed vertical line denotes the implementation of new strategies at the start of 2018.

References

1. Simarro PP, Cecchi G, Franco JR, et al. Monitoring the Progress towards the Elimination of Gambiense Human African Trypanosomiasis. PLoS Neglected Tropical Diseases **2015**; 9(6): e0003785-14.
2. Simarro PP, Cecchi G, Franco JR, et al. Estimating and mapping the population at risk of sleeping sickness. PLoS Neglected Tropical Diseases **2012**; 6(10): e1859-e.
3. Simarro PP, Cecchi G, Paone M, et al. The Atlas of human African trypanosomiasis: a contribution to global mapping of neglected tropical diseases. International Journal of Health Geographics **2010**; 9(1): 57.
4. Stone CM, Chitnis N. Implications of Heterogeneous Biting Exposure and Animal Hosts on *Trypanosomiasis brucei gambiense* Transmission and Control. PLoS Computational Biology **2015**; 11(10): e1004514-22.

5. Rock KS, Pandey A, Ndeffo-Mbah ML, et al. Data-driven models to predict the elimination of sleeping sickness in former Equateur province of DRC. Epidemics, **2017**:1-22.
6. Rock KS, Torr SJ, Lumbala C, Keeling MJ. Quantitative evaluation of the strategy to eliminate human African trypanosomiasis in the Democratic Republic of Congo. **2015**; 8(1): 1-13.
7. Rock KS, Torr SJ, Lumbala C, Keeling MJ. Predicting the Impact of Intervention Strategies for Sleeping Sickness in Two High-Endemicity Health Zones of the Democratic Republic of Congo. PLoS Neglected Tropical Diseases **2017**; 11(1): e0005162.
8. Mahamat MH, Peka M, Rayaisse JB, et al. Adding tsetse control to medical activities contributes to decreasing transmission of sleeping sickness in the Mandoul focus (Chad). PLoS Neglected Tropical Diseases **2017**; 11(7): e0005792.
9. Pandey A, Atkins KE, Bucheton B, et al. Evaluating long-term effectiveness of sleeping sickness control measures in Guinea. Parasites & Vectors **2015**; 8(1): 550.
